# *PRDM8* reveals aberrant DNA methylation in aging syndromes and is relevant for hematopoietic and neuronal differentiation

**DOI:** 10.1186/s13148-020-00914-5

**Published:** 2020-08-20

**Authors:** Olivia Cypris, Monika Eipel, Julia Franzen, Corinna Rösseler, Vithurithra Tharmapalan, Chao-Chung Kuo, Margherita Vieri, Miloš Nikolić, Martin Kirschner, Tim H. Brümmendorf, Martin Zenke, Angelika Lampert, Fabian Beier, Wolfgang Wagner

**Affiliations:** 1 Helmholtz-Institute for Biomedical Engineering, Stem Cell Biology and Cellular Engineering, RWTH Aachen University, Pauwelsstrasse 20, Aachen, Germany; 2 Institute of Physiology, Medical Faculty University Hospital Aachen, RWTH Aachen University, Aachen, Germany; 3 Department of Hematology, Oncology, Hemostaseology and Stem Cell Transplantation, Medical Faculty University Hospital Aachen, RWTH Aachen University, Aachen, Germany; 4 Institute for Biomedical Engineering – Cell Biology, RWTH Aachen University Medical School, Aachen, Germany

**Keywords:** PRDM8, Epigenetic clock, DNA methylation, Telomere, Aging, Dyskeratosis congenita, Aplastic anemia, iPSC, Hematopoietic differentiation, Neuronal differentiation

## Abstract

**Background:**

Dyskeratosis congenita (DKC) and idiopathic aplastic anemia (AA) are bone marrow failure syndromes that share characteristics of premature aging with severe telomere attrition. Aging is also reflected by DNA methylation changes, which can be utilized to predict donor age. There is evidence that such epigenetic age predictions are accelerated in premature aging syndromes, but it is yet unclear how this is related to telomere length. DNA methylation analysis may support diagnosis of DKC and AA, which still remains a challenge for these rare diseases.

**Results:**

In this study, we analyzed blood samples of 70 AA and 18 DKC patients to demonstrate that their epigenetic age predictions are overall increased, albeit not directly correlated with telomere length. Aberrant DNA methylation was observed in the gene *PRDM8* in DKC and AA as well as in other diseases with premature aging phenotype, such as Down syndrome and Hutchinson-Gilford-Progeria syndrome. Aberrant DNA methylation patterns were particularly found within subsets of cell populations in DKC and AA samples as measured with barcoded bisulfite amplicon sequencing (BBA-seq). To gain insight into the functional relevance of PRDM8, we used CRISPR/Cas9 technology to generate induced pluripotent stem cells (iPSCs) with heterozygous and homozygous knockout. Loss of PRDM8 impaired hematopoietic and neuronal differentiation of iPSCs, even in the heterozygous knockout clone, but it did not impact on epigenetic age.

**Conclusion:**

Taken together, our results demonstrate that epigenetic aging is accelerated in DKC and AA, independent from telomere attrition. Furthermore, aberrant DNA methylation in *PRDM8* provides another biomarker for bone marrow failure syndromes and modulation of this gene in cellular subsets may be related to the hematopoietic and neuronal phenotypes observed in premature aging syndromes.

**Graphical abstract:**

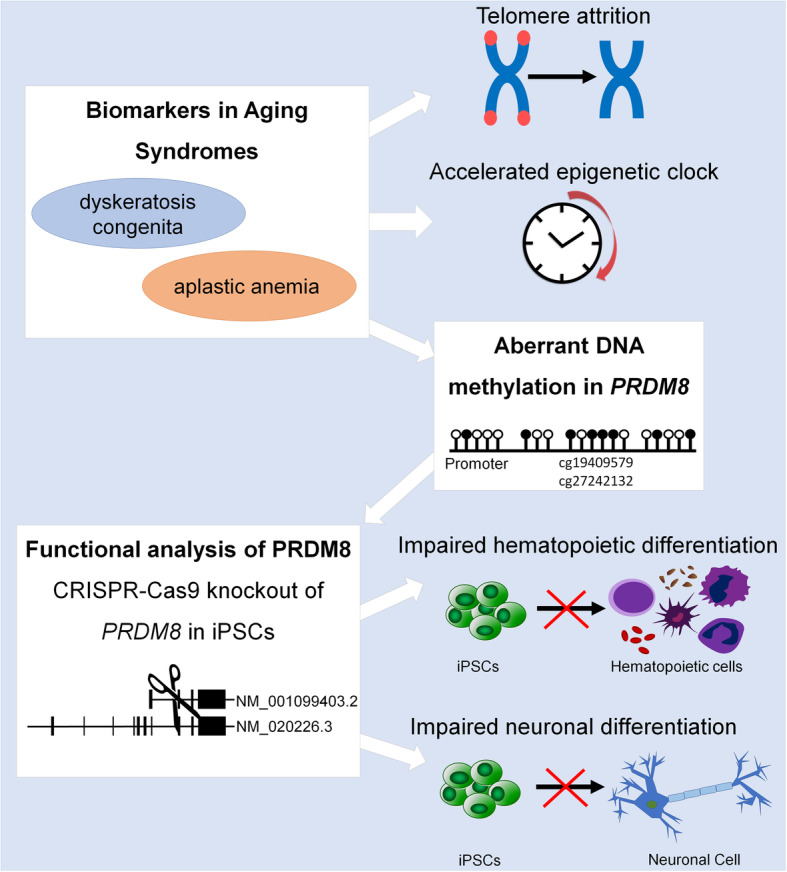

## Background

Premature aging syndromes, such as Down syndrome, Werner syndrome, or dyskeratosis congenita (DKC), are often associated with impaired neurologic development or hematological abnormalities [1–3]. Down syndrome is caused by trisomy 21, Werner syndrome results from mutations in the Werner syndrome ATP-dependent helicase (*WRN*), and DKC is a paradigmatic disease for studying the consequences of critical short telomeres [1, 2]. Premature telomere shortening in DKC is caused by mutations in genes of the telomerase complex such as telomerase reverse transcriptase (*TERT*) or telomerase RNA component (*TERC*), resulting in impaired telomere maintenance [4]. Clinically, DKC patients show a triad of skin hyperpigmentation, nail dystrophy, and oral leukoplakia [1, 5]. Severe forms of DKC are characterized by a neurological impairment such as disturbed cerebellar development, whereas cryptic forms develop slowly without manifestation of characteristic symptoms before adulthood and may develop bone marrow failure, which can be observed in up to 90% of all DKC patients by the age of 30 [1, 6, 7]. The bone marrow is then hypocellular and indistinguishable from patients with acquired aplastic anemia (AA), a disease characterized by a T cell-mediated autoimmune destruction of the hematopoietic stem cell compartment [8, 9]. Diagnosis of DKC is often based on premature telomere shortening in peripheral blood leukocytes compared to healthy individuals, followed by genetic analysis of the relevant telomere maintenance-associated genes [10]. Of note, in acquired AA, telomere length in the granulocyte compartment can also show substantial attrition and was shown to reflect the degree of autoimmune-mediated damage to the hematopoietic stem cell compartment [11]. This may hamper correct diagnosis of DKC [12], which is of utmost importance since DKC, in contrast to acquired AA, is not responding to immunosuppressive therapy and requires optimized conditioning protocols in case of allogeneic stem cell transplantation [13].

Another hallmark of aging, besides telomere shortening, is epigenetic changes. Over the life time, the DNA methylation pattern changes continuously at specific CG dinucleotides (CpG sites) in the genome. Analysis of such age-associated DNA methylation changes can be used as a biomarker for aging, often referred to as “epigenetic clock” [14]. Various different epigenetic signatures have been described, which were often based on DNA methylation profiles with Illumina BeadChip technology [15, 16]. For more cost-effective high throughput analysis, we derived a targeted epigenetic age-predictor based on three age-associated CpG sites, which was specifically trained for blood [17]. In a preliminary analysis of 5 DKC and 15 AA patients, we observed that this epigenetic clock might be accelerated in bone marrow failure syndromes [17]. However, while DNA methylation profiles of three DKC patients in a further study did not reveal unequivocal acceleration of epigenetic age, we did observe a significant hypermethylation within the promoter region of the short transcript of the PR Domain Zinc Finger Protein 8 (*PRDM8*) [18]. PRDM8 belongs to the PRDM family characterized by a conserved N-terminal PR domain related to the catalytic SET domains, which represent a large group of histone methyltransferases [19–22] that seem to be generally involved in regulation of stem cell function, developmental processes, and malignant transformation [23, 24].

In this study, we demonstrate that epigenetic aging is overall moderately accelerated in DKC and AA, and this is independent from telomere attrition. Furthermore, DKC and AA revealed aberrant DNA methylation within the gene *PRDM8*, which was also observed for other premature aging syndromes. To gain further insight into the biological function of this gene in induced pluripotent stem cells (iPSCs), we created *PRDM8* knockout clones, resulting in impaired hematopoietic and neuronal differentiation.

## Results

### Telomere attrition and accelerated epigenetic aging in bone marrow failure syndromes

**Fig. 1:**
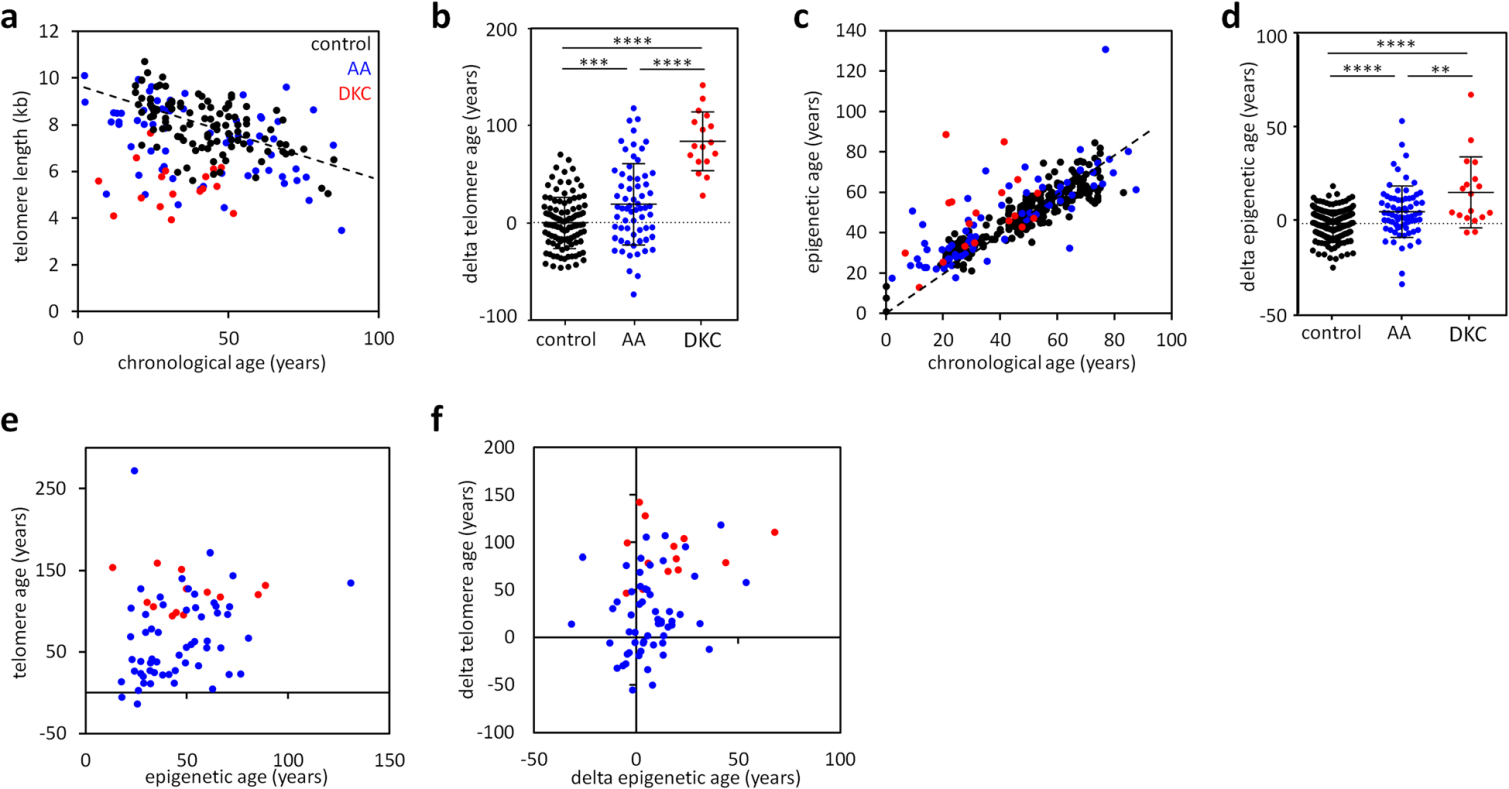
Telomere age and epigenetic age are increased in dyskeratosis congenita and aplastic anemia. **a** Telomere lengths of 105 healthy donors [17], 65 aplastic anemia (AA), and 17 dyskeratosis congenita (DKC) patients were measured in granulocytes and correlated to chronological age. DKC and AA patients show a reduced telomere length compared to healthy controls. **b** Offset of predicted telomere age (delta age) was higher for DKC and AA patients than for healthy controls [17]. *t* test: *** *P < 0.001*, **** *P* < 0.0001. **c** Epigenetic age predictions of 70 AA and 18 DKC samples revealed much lower correlation to chronological age than 243 healthy controls [17, 25]. **d** The difference between predicted epigenetic age and chronological age (delta age) was higher for DKC and AA than for healthy controls, as described for other samples before [17]. *t* test: ** *P* < 0.01, **** *P* < 0.0001. **e**, **f** Telomere age and epigenetic age (**e**), as well as delta telomere age and delta epigenetic age (**f**), do not correlate in 62 AA and 13 DKC samples

Telomere length was measured in granulocytes of 65 AA and 17 DKC patients. All DKC patients and most AA patients revealed significant telomere attrition (Fig. 1a). In comparison to the age-adjusted linear model of the 105 healthy donors [17], the predicted age based on telomere length in granulocytes revealed a significant offset (delta telomere age) in AA (mean absolute error [MAE] = 35.03 years; *P* = 0.0003), which were on average predicted 19.05 years older than their chronological age, and DKC (MAE = 84.14 years; *P* < 0.0001; Fig 1b), which were also on average predicted 84.14 years older than their chronological age. Similar results were observed when telomere length was analyzed in lymphocytes (Additional file 1: Fig. S1a, b). This is in line with previous observations on 11 independent DKC and 27 independent AA samples [18].

We then analyzed epigenetic age in these samples using our previously described epigenetic aging signature based on DNA methylation at three CpGs within the genes phosphodiesterase 4C (*PDE4C*; CpG next to cg17861230, which is not presented by the Illumina BeadChip), integrin alpha 2b (*ITGA2B*; cg25809905), and aspartoacylase (*ASPA*; cg02228185) [17]. For blood samples of 243 healthy controls [17, 25], we observed a good correlation with chronological age (MAE = 5.45 years; *R*^2^ = 0.83), whereas there was a significant offset in epigenetic age predictions for the 70 AA (MAE = 10.50 years; *R*^2^ = 0.65; *P* < 0.0001) and 18 DKC patients (MAE = 17.38 years; *R*^2^ = 0.15; *P* < 0.0001). On average, AA and DKC samples were predicted 6.06 years, and 16.36 years older than their chronological age (Fig. 1c, d), which is in line with previous observations [18]. We then analyzed if telomere attrition was correlated with accelerated epigenetic aging, but this was not the case (Fig. 1e, f and Additional file 1: Fig. S1c, d). Thus, telomere attrition and epigenetic aging seem to reflect two independent processes of premature cellular aging in these bone marrow failure syndromes.

### Aberrant DNA methylation patterns within the gene *PRDM8* in AA and DKC

**Fig. 2:**
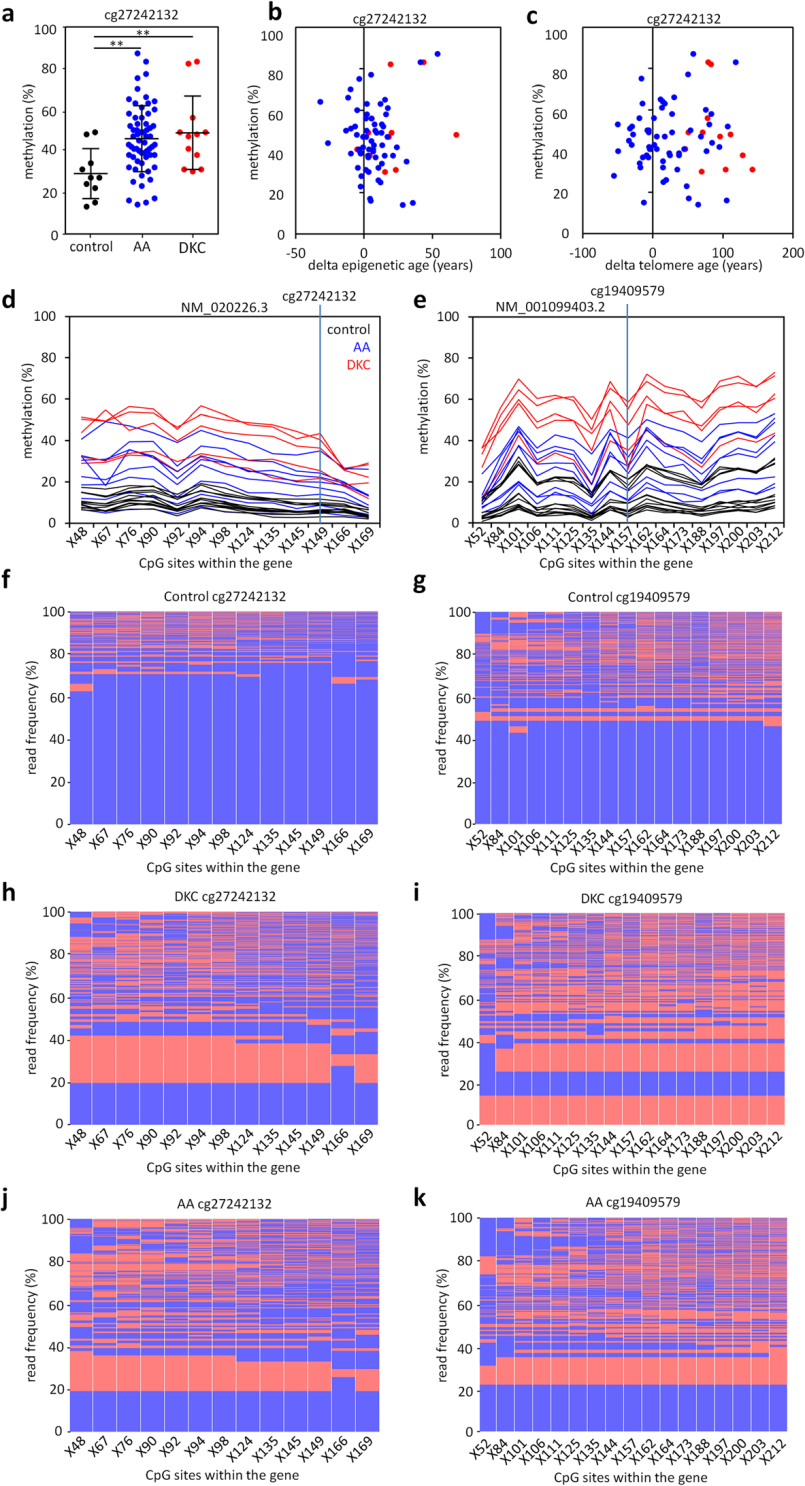
Hypermethylation in *PRDM8* in dyskeratosis congenita and aplastic anemia. **a** DNA methylation was measured by MassARRAY at the CpG site cg27242132 in blood samples of 62 new aplastic anemia (AA) and 12 new dyskeratosis congenita (DKC) patients, as compared to 10 previously described healthy controls [18]. *t* test: ** *P* < 0.01. **b**, **c** DNA methylation in cg27242132 does not correlate to epigenetic age (**b**) or telomere age (**c**). **d**, **e** Barcoded bisulfite amplicon sequencing (BBA-seq) was used to determined DNA methylation around the two relevant CpGs (cg27242132 and cg19409579) in independent samples: 12 controls, 8 AA, and 5 DKC validated higher methylation in DKC and AA compared to controls across the amplicons with 13 (assay 1) and 17 (assay 2) neighboring CpGs. **f**–**k** The frequencies of DNA methylation patterns within individual BBA-seq reads are exemplarily depicted for both *PRDM8* amplicons in a healthy donor (**f**, **g**), a DKC patient (**h**, **i**) and a AA patient (**j**, **k**)

The gene of PR Domain Zinc Finger Protein 8 (*PRDM8*) was previously shown to be hypermethylated in DKC and AA patients [18]. In continuation of this work, we analyzed if DNA methylation at this gene is a suitable biomarker for these bone marrow failure syndromes. In fact, two independent DNA methylation assays for two CpGs (cg19409579 and cg27242132) demonstrated that the majority of AA and DKC samples revealed significantly higher DNA methylation as compared to blood samples of healthy controls (Fig. 2a and Additional file 1: Fig. S2a). Hypermethylation at *PRDM8* did not correlate with accelerated epigenetic age or telomere attrition, indicating that these processes are not directly associated with each other (Fig. 2b, c and Additional file 1: Fig. S2b, c). Furthermore, there was no correlation of DNA methylation in the *PRDM8* assays with blood counts (Additional file 1: Fig. S3a, b).

The region in *PRDM8* is rich in CpG sites and therefore these measurements were performed with MassARRAY technology to facilitate analysis of longer amplicons than allowed for pyrosequencing. However, both methods only provide average DNA methylation levels across all cells within a sample. Therefore, we utilized barcoded bisulfite amplicon sequencing (BBA-seq) to obtain individual reads by deep sequencing that reflect the heterogeneity of DNA methylation patterns within the cells of a given sample. We exemplarily analyzed 12 healthy, 8 AA, and 5 DKC samples that were not included in the above analysis with BBA-seq. For both *PRDM8* regions (around cg27242132 and cg19409579), the results clearly validated aberrant DNA methylation within 13 (assay 1) and 17 (assay 2) neighboring CpG sites (Fig. 2d, e). We then analyzed the DNA methylation patterns within individual BBA-seq reads of single DNA strands. In healthy samples, we observed that the CpGs within these regions were predominately non-methylated, whereas about 25 to 60% of the reads provided stochastic patterns of DNA methylation at neighboring CpGs (Fig. 2f, g). The fraction of methylated reads was higher in AA and DKC samples, and many of them comprised reads that were consistently methylated across all CpGs (Fig. 2 h–k). There was no clear difference in the DNA methylation patterns of AA and DKC—while the DNA methylation level was overall higher in these samples, they also comprised entirely non-methylated strands, reads with stochastic DNA methylation at neighboring CpGs, and often reads that are consistently methylated.

### Aberrant DNA methylation in *PRDM8* is not reflected on average gene expression levels

**Fig. 3:**
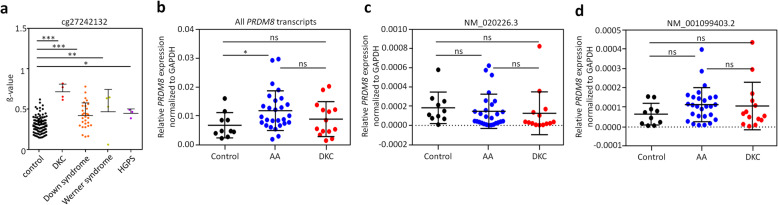
DNA methylation in *PRDM8* in premature aging syndromes and differential gene expression. **a** To evaluate aberrant DNA methylation in *PRDM8* (at cg27242132) in other premature aging syndromes, we used available DNA methylation profiles of 103 healthy controls (GSE36054, GSE32148, GSE49064), 4 DKC samples (GSE75310), 29 Down syndrome samples (GSE52588), 4 Werner syndrome samples (GSE42865), and 3 Hutchinson-Gilford-Progeria syndrome (HGPS) samples (GSE42865). DNA methylation levels (*β* values) revealed hypermethylation in all premature aging syndromes. *t* test: * *P* < 0.05, ** *P* < 0.01, *** *P* < 0.001. **b**–**d** Expression of all *PRDM8* transcripts (**b**), the long *PRDM8* transcript (NM020026.3; **c**), and the short transcript (NM001099403.2; **d**) was analyzed by qRT-PCR in 10 controls, 27 AA, and 14 DKC patients. *t* test: * *P* < 0.05; ns, not significant

Since DKC is one of various prominent premature aging syndromes, we subsequently analyzed if *PRDM8* is also aberrantly methylated in other premature aging syndromes. To this end, we utilized DNA methylation profiles of Werner syndrome (GSE42865), Hutchinson-Gilford-Progeria syndrome (HGPS, GSE42865), Down syndrome (GSE52588), and profiles of DKC patients (GSE75310). Aberrant hypermethylation of the two relevant CpG sites cg19409579 and cg27242132 was particularly observed in DKC, while there was also some offset in other premature aging diseases (Fig. 3a and Additional file 1: Fig. S4a). Furthermore, aberrant hypomethylation within the promoter region of the longer of the two *PRDM8* transcripts (NM_020226.3) was observed in all premature aging syndromes, albeit this was less prominent in DKC and Werner syndrome (Additional file 1: Fig. S4b). These results indicate that aberrant epigenetic patterns in *PRDM8* are frequently observed in premature aging syndromes.

We have subsequently analyzed if *PRDM8* gene expression is also aberrantly regulated in these syndromes. Our previous analysis with quantitative RT-PCR revealed downregulation of *PRDM8* in five DKC samples [18]. We have now performed qRT-PCR for a new set of more patient samples (10 healthy controls, 27 AA patients, and 14 DKC patients) and did not observe a significant difference in gene expression levels of either the long (NM_020226.3) or the short transcript of *PRDM8* (NM_001099403.2; Fig. 3b–d). Gene expression levels for all transcripts were slightly higher in AA than in controls, but expression was generally rather low in the blood samples. In addition, we have analyzed public gene expression profiles of Down syndrome (blood; GSE35665, *n* = 15 [26]), Werner syndrome (fibroblasts; GSE48761, *n* = 10 [27]), and HGPS (fibroblasts; GSE69391, *n* = 6 [28] and GSE3860, *n* = 3 [29]), but none of these datasets revealed significant gene expression changes in *PRDM8* (data not shown). This finding might be attributed to the fact that *PRDM8* was also hardly expressed in those datasets. Furthermore, as suggested by the BBA-seq data, aberrant DNA methylation patterns are not observed in all DNA molecules of a sample and hence a potential epigenetic effect might be masked by other cell populations without differential DNA methylation. Either way, *PRDM8* expression might be relevant during cellular differentiation or in specific cellular subsets.

### *PRDM8* knockout impairs hematopoietic differentiation

**Fig. 4:**
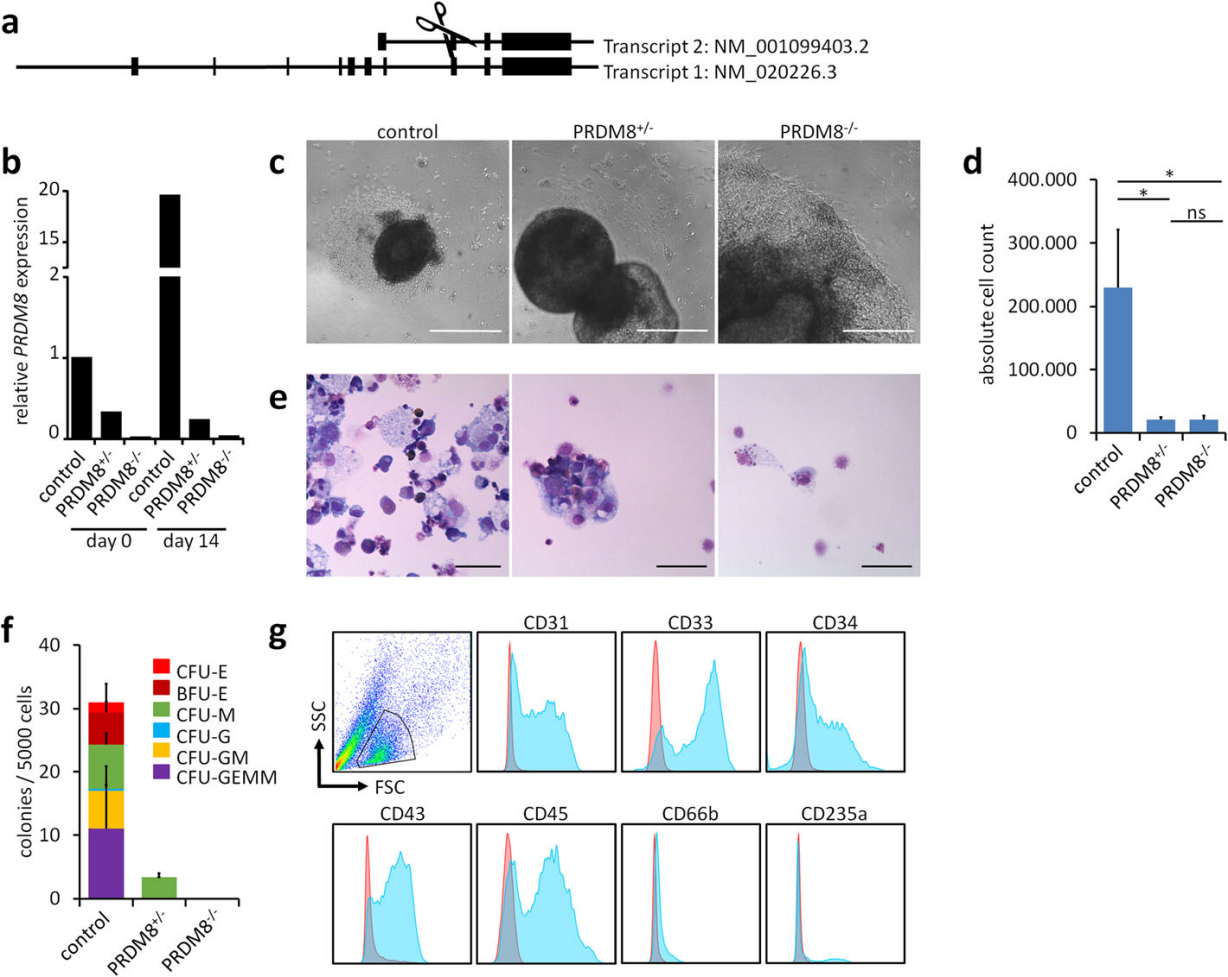
Impaired hematopoietic differentiation of *PRDM8*^−/−^ iPSCs. **a** Scheme of two main transcripts of *PRDM8* (NM_020226.3 and NM_001099403.2) and sites of genomic editing. Two pairs of guide RNAs (gRNAs) were designed targeting the intron/exon boundary at the start codon of both transcripts. **b** Genome editing was confirmed by gene expression analysis after 14 days of embryoid body assay (normalized to *GAPDH* and *PRDM8* expression in the undifferentiated control cells). **c** Phase contrast pictures of EBs after 16 days of hematopoietic differentiation. The control EB produces hematopoietic progenitor cells, whereas this is not the case for the *PRDM8*^*+/*−^ clone and the *PRDM8*^−*/*−^ clone, which consistently revealed enhanced growth. Scale bar, 500 μM. **d** Knockout of *PRDM8* resulted in a significantly lower number of hematopoietic progenitor cells. *t* test: * *P* < 0.05; ns, not significant. **e** Cytospins supported impaired hematopoiesis after *PRDM8* knockout. Scale bar, 500 μM. **f** The colony forming unit (CFU) potential is lost in *PRDM8* knockout clones. **g** Flow cytometry substantiates hematopoietic differentiation of the control iPSCs (read line: autofluorescence; blue line: with antibodies)

To gain insight into the functional relevance of PRDM8, we generated clonal induced pluripotent stem cell (iPSC) lines with homozygous and heterozygous gene knockout of *PRDM8*. Deletion of the start codon with CRISPR/Cas9 nickase resulted in loss of PRDM8 (Fig. 4a). Sequencing indicated that deletion of the intron/exon boundary led to a reading frame shift and formation of a premature stop codon, thus generating a complete knockout of the PRDM8 protein. The *PRDM8*^+/−^ and *PRDM8*^−/−^ iPSCs maintained expression of the pluripotency markers OCT4 and TRA-1-60, they could be culture expanded over many passages (up to 60 passages), and they revealed a positive Epi-Pluri-Score [30], indicating that *PRDM8* knockout clones remained pluripotent (Additional file 1: Fig. S5a, b). Furthermore, upon differentiation in embryoid bodies (EBs) for 2 weeks, qRT-PCR analysis validated upregulation of marker genes for ectodermal, mesodermal, and endodermal lineages, albeit upregulation of the neuronal markers nestin (*NES*) and paired box 6 (*PAX6*) was reduced for the *PRDM8* knockouts (Additional file 1: Fig. S5c). Expression of *PRDM8* was hardly detectable in the knockout cells by qRT-PCR upon 14 days of differentiation in the embryoid body assay (Fig. 4b).

We have then differentiated three iPSC lines, including the isogenic iPSC line from which we generated the knockout clones, as well as the *PRDM8*^+/−^ and *PRDM8*^−/−^ iPSCs toward the hematopoietic lineage using an EB-based differentiation protocol [31]. After 16 days, we observed production of hematopoietic cells from the EBs in control iPSCs. In contrast, hematopoietic differentiation was hardly observed with the *PRDM8*^+/−^ and particularly the *PRDM8*^−/−^ iPSCs and their EBs acquired a much larger size than EBs of control iPSCs (Fig. 4c, d). The few iPSC-derived hematopoietic cells of the knockout clones did not reveal typical hematopoietic morphology in cytospins (Fig. 4e). Furthermore, colony formation potential was lost upon *PRDM8* knockout (Fig. 4f and Additional file 1: Fig. S6). *PRDM8*^+/−^ and *PRDM8*^−/−^ iPSCs did not produce enough progenitor cells for flow cytometry analysis, but for control iPSC expression of hematopoietic surface markers was confirmed (Fig. 4g). Thus, control iPSCs revealed clear hematopoietic differentiation, whereas differentiation was abrogated upon *PRDM8* knockout.

### *PRDM8* knockout impairs neuronal differentiation

**Fig. 5:**
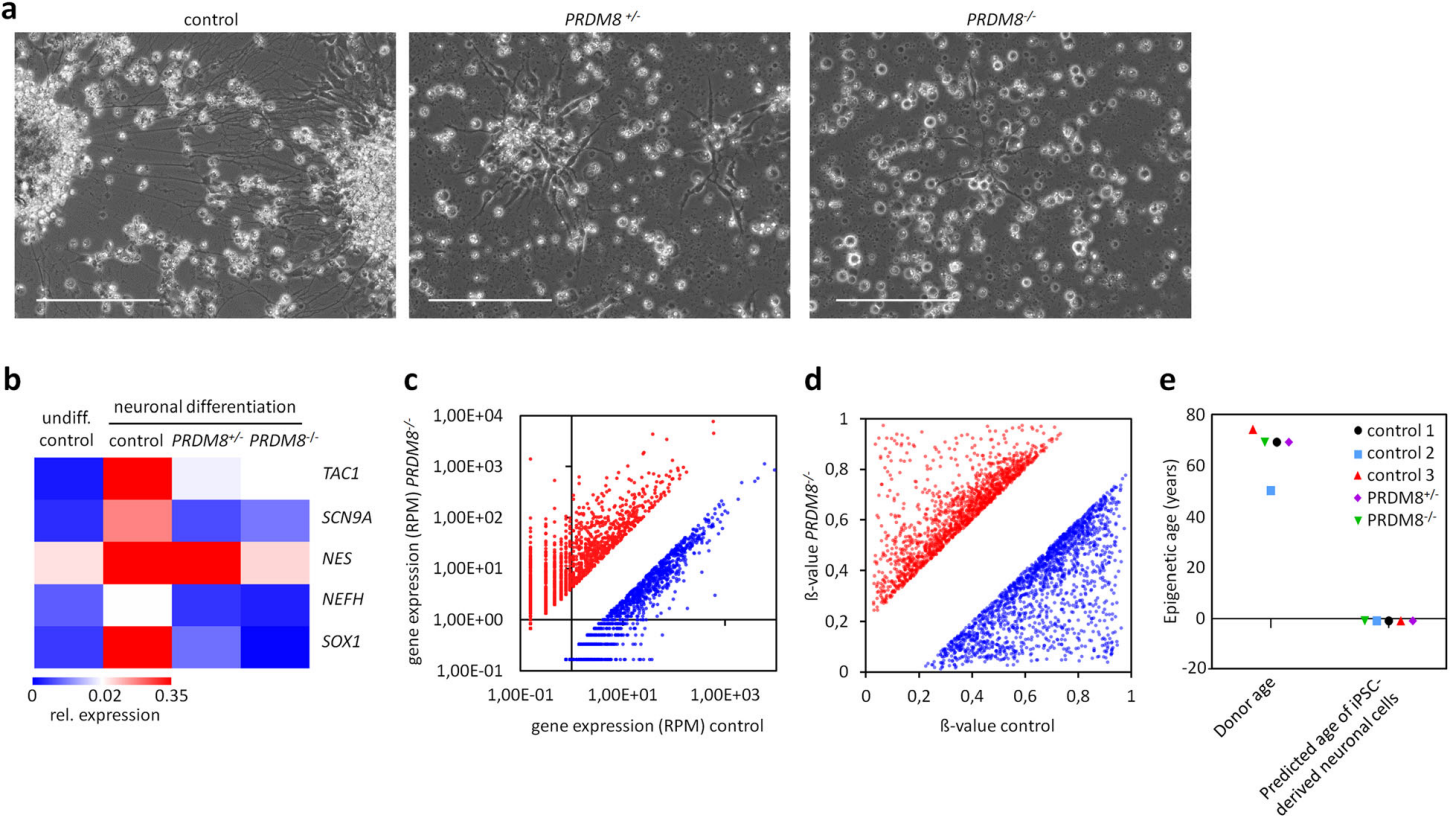
*PRDM8* knock out leads to impaired neuronal differentiation. **a** Representative phase contrast pictures of neuronal differentiations on day 13. Control cells reveal typical aggregates of immature sensory neurons, which are absent in the *PRDM8* knockout cells. Scale bar, 200 μM. **b** Quantitative RT-PCR analysis after 10 days of neuronal differentiation shows upregulation of neuronal markers in control cells, but not in *PRDM8* knockouts (color code depicts mean fold change versus *GAPDH*). **c** Gene expression changes in RNA-Seq after 27 days of peripheral neuron differentiation (RPM, reads per million; genes with a log2 fold change above 2 or below − 2 are depicted). **d** CpG sites that are either 20% hypermethylated (red, 1511 CpGs) or hypomethylated (blue, 1738 CpGs) in the *PRDM8*^−/−^ clone compared to the isogenic control. **e** Epigenetic age of control and knockout iPSCs after neuronal differentiation was close to 0 year.

We then differentiated iPSCs toward the neuronal lineage because PRDM8 was reported to be involved in neuronal development in mice [32, 33]. After 2 weeks of neuronal differentiation, microscopic analyses revealed typical ganglion-like structures with bridging neurites in the control cells. In contrast, these neuronal agglomerates were hardly observed in the derivatives of *PRDM8*^+/−^ and *PRDM8*^−*/*−^ iPSCs (Fig. 5a). Furthermore, after 10 days of neuronal differentiation, qRT-PCR analysis of the neuronal marker genes tachykinin precursor 1 (*TAC1*), voltage-gated sodium channel subunit alpha (*SCN9A*), nestin (*NES*), neurofilament heavy polypeptide (*NEFH*), and SRY-box transcription factor 1 (*SOX1*) revealed clear upregulation in the isogenic iPSC control, whereas this was not observed in the *PRDM8*^+/−^ and *PRDM8*^−/−^ iPSCs (Fig. 5b). We then compared global gene expression profiles of the syngenic control and *PRDM8*^−*/*−^ iPSCs upon neuronal differentiation: in the *PRDM8*^−/−^ cells, 1280 genes were more than 4-fold less expressed, whereas 1769 genes were at least 4-fold higher expressed than in controls (Fig. 5c; Additional file 2: Table S1). These genes were particularly associated with gene ontology categories for cell adhesion and neuronal development (Additional file 1: Fig. S7a). Overall, these results clearly support the notion that loss of PRDM8 impairs neuronal differentiation of iPSCs.

### *PRDM8* knockout does not impact on epigenetic age

Subsequently, we analyzed DNA methylation profiles of a syngenic control clone and the *PRDM8*^−/−^ iPSC clone upon 10 days of neuronal differentiation with the Illumina Infinium MethylationEPIC BeadChip. Filtering for CpGs with more than 20% change in DNA methylation levels between control and knockout cells showed that 1738 CpGs were hypomethylated and 1511 CpGs were hypermethylated in *PRDM8*^−/−^ versus control (Fig. 5d; Additional file 3: Table S2). We then analyzed if loss of PRDM8 significantly accelerated the epigenetic clock in iPSC-derived neuronal cells. Here, we used the epigenetic clock by Horvath [34] because it was trained on multiple different cell types, while our 3 CpG age predictor utilized for targeted analysis of DKC and AA samples was specifically trained for blood. There was no difference in epigenetic age predictions upon 10 days of neuronal differentiation between three control lines, *PRDM8*^+/−^, and *PRDM8*^−/−^ clones (Fig. 5e). In fact, all iPSC-derived cell preparation was estimated close to 0 year, indicating that loss of PRDM8 does not clearly accelerate the epigenetic clock in our iPSC model system.

## Discussion

Diagnosis of bone marrow failure syndromes remains a challenge. In this study, we further validated that leukocytes of DKC and AA patients reveal telomere attrition and accelerated epigenetic aging. Moderate acceleration of epigenetic aging has also been described for Down syndrome [34], HGPS [35], or Werner syndrome [36]. Notably, telomere attrition and epigenetic aging seem to be independent, which is in line with previous studies [37–40]. It should be noted that epigenetic age can be influenced by various external parameters, such as gender, alcohol consumption, or body mass index [17]. However, the prominent increase in epigenetic age in AA and DKC patients argues for a direct effect by premature aging. Therefore, analysis of epigenetic age, in addition to telomere length analysis, can provide added value to identify DKC and AA patients because not all patients revealed shortened telomeres or disease-associated mutations [41].

Furthermore, we analyzed DNA methylation within *PRDM8* in 62 independent AA and 13 independent DKC samples with MassARRAY and could confirm that this region frequently reveals aberrant hypermethylation. This was further validated with BBA-seq in 8 additional AA and 5 DKC samples. Thus, we provide a new deep-sequencing-based assay that may be better applicable by other scientists and that facilitates longer amplicons. Furthermore, the individual reads provide insight into the DNA methylation pattern: aberrant gain of DNA methylation seems to be increased in cellular subsets, but the neighboring CpGs do not always appear to be coherently modified, as might be expected by binding of DNA methyltransferases (e.g., DNMT3A or DNMT3B) to a specific DNA strand. On the other hand, there was no evidence that DNA methylation in *PRDM8* was associated with blood counts. In future studies, it will be interesting to better understand how the aberrant and heterogeneous DNA methylation within *PRDM8* is evoked and controlled.

Notably, genome wide DNA methylation profiles of other premature aging syndromes provided similar hypermethylation within *PRDM8* and hypomethylation in the promoter of the long transcript of *PRDM8*. In fact, one of the top 25 differentially methylated regions (DMRs) reported in Werner syndrome is located within *PRDM8* [42]. It is generally anticipated that aberrant DNA methylation is accompanied with changes in gene expression. However, our qRT-PCR experiments of AA and DKC did not reveal significant downregulation of either the short or the long transcript of *PRDM8.* Furthermore, we did not observe significant changes in gene expression of *PRDM8* in datasets of premature aging syndromes. This might be partly attributed to the fact that *PRDM8* is hardly expressed in blood and fibroblasts. Moreover, as reflected by the BBA-seq results, the aberrant hypermethylation in *PRDM8* was not consistent across all DNA molecules of a sample and this might occlude differential gene expression in specific subpopulations. A transient expression of *PRDM8* might still be relevant for regulation of developmental processes or premature aging phenotypes, even if the gene is hardly expressed in the adult tissues. However, this functional link cannot be directly proven by our analysis.

So far, little is known about the biological function of PRDM8—particularly in humans. It has been suggested that different isoforms of PRDM proteins (with or without the PR domain) play opposing roles in malignancies: The longer transcript was suggested to function as tumor suppressor, whereas the shorter variant acts as an oncogene [43]. In the murine model system, it was reported that *Prdm8* is expressed in brain tissues [33, 44]. Prdm8 seems to function as a repressor for Cadherin-11 to ensure proper neural circuit formation [32]. Inoue et al. described that *Prdm8* knockout mice reveal growth retardation and abnormal generation of upper-layer neocortical neurons [33]. When analyzing human iPSCs with *PRDM8* knockout, we observed impaired neural differentiation potential—even for the heterozygous knockout iPSCs. This may indicate that aberrant regulation of PRDM8 may contribute to impaired neurological development, which is characteristic for many premature aging syndromes [45].

In addition, iPSCs with *PRDM8* knockout revealed impaired hematopoietic differentiation—another key feature of premature aging syndromes. Other PRDM family members are also involved in hematopoiesis and leukemic transformation. For example, PRDM1 seems to be involved in differentiation, maintenance and function of different myeloid cell types [46], B cells [47], T cells [48], and NK cells [49]. Loss of Prdm3 results in a decreased hematopoietic stem cell pool [50, 51]. PRDM8 and PRDM16 might have partly redundant function. Both of them seem to be involved in methylation of lysine 9 of histone 3 (H3K9) [52, 53], a histone mark that is tightly associated with the DNA methylation pattern. Prdm16 was shown to be critical for establishing and maintaining hematopoietic stem cells and it is aberrantly expressed in AML [54–56]. Furthermore, Prdm16 is also an important factor for neuronal stem cells [55] and, like Prdm8, plays a role in neocortical development [57]. Notably, in our previous work, we demonstrated that not only PRDM8, but also PRDM16, is aberrantly methylated in DKC [18], and thus both of them might be relevant for the pathophysiology of the disease.

## Conclusions

Diagnosis of AA and DKC patients remains a challenge. Our results support the notion that both premature aging syndromes frequently reveal telomere attrition, accelerated epigenetic aging, and aberrant DNA methylation in *PRDM8.* There is little correlation between these biomarkers. Therefore, analysis of epigenetic age or aberrant DNA methylation in *PRDM8* might be advantageous in patients without significant telomere attrition or specific mutations to identify these bone marrow failure syndromes. In the future, it will be important to also consider samples with other differential diagnoses to better define the specificity and sensitivity of our assays. BBA-seq analysis for *PRDM8* and epigenetic aging [25] can provide insight into the heterogeneity of aberrant DNA methylation within a sample and it will be interesting to better understand how these processes are regulated. While the functional relevance of aberrant DNA methylation in *PRDM8* needs to be further explored, our results indicate that the gene plays an important role for hematopoietic and neuronal differentiation. Thus, it might contribute to the phenotype of premature aging syndromes, albeit the functional link remains to be demonstrated.

## Methods

### Sample collection and next generation sequencing (NGS)

Blood samples were obtained from the Registry for Telomeropathies and Aplastic Syndromes of RWTH Aachen University and participating hospitals. The study was approved by the local ethic committee and all samples were taken after written consent (EK206/09). All DKC patients revealed lymphocyte telomere length below 1% percentile and diagnosis was complemented by clinical and genotypic characteristics [12, 58]. All AA and DKC samples were screened by NGS using a self-customized gene panel containing the entire coding sequence of genes that are known to play a relevant role in DKC (*CTC1*, *DKC1*, *NHP2*, *NOP10*, *RTEL1*, *TERC*, *TERT*, *TCAB1*, *USB1*, and exon 6 of *TINF2*, which is a known hot spot region) [12]. Library preparation and sequencing were performed with the TruSeq Custom Amplicon Kit and the MiSeq Reagent Kit v2 (all from Illumina) using a MiSeq Illumina platform. Sequencing was performed with 250 bp paired end and data was first analyzed with the Illumina RTA software. Afterwards, the SeqNEXT software (version 4.3.0, JSI medical systems GmbH, Ettenheim, Germany) was used for alignment and variant calling. A mean coverage of 100 × was reached and since we looked for germline variants, a cutoff of mutant allele frequency of > 30% or ≥ 10 absolute mutant reads was chosen. Further information about the patients is provided in Additional file 4: Table S3.

### Analysis of telomere length and telomere age prediction

Telomere length of granulocytes and lymphocytes was analyzed in 105 samples from healthy donors as described before [17], 70 independent patients with AA, and 18 independent patients with DKC. In 5 AA patients and 1 DKC patient, telomere length measurement of the granulocytes was not possible due to insufficient cell number. Flow-FISH for telomere length was performed as described in detail before [12, 59]. In brief, samples were mixed with a FITC-labeled or Alexa488-labled telomere-specific (CCCTAA)3-peptide nucleic acid FISH probe (Panagene, Daejeon, South Korea) for DNA hybridization followed by DNA counterstaining with LDS 751 (Sigma Aldrich, St. Louis, MO, USA). An FC 500 flow cytometer (Becton Dickinson, Franklin Lakes, NJ, USA) was used for data acquisition. Bovine thymocytes were used as internal controls to calculate telomere length in kilobases and samples were measured in triplicates. The cow thymocytes as well as granulocytes and lymphocytes from human samples were identified based on forward scatter and LDS 751 binding to double-stranded DNA. Telomere age was estimated by linear regression on the age-adjusted samples from healthy donors. Mean average error [MAE] was calculated as follows:

$$ \mathrm{MAE}=\frac{1}{n}\ {\sum}_{i=1}^n\left|{x}_i\right| $$

with *x* = delta age. Mean delta age ($\overline{x}$) was calculated as follows:

$$ \overline{x}=\frac{1}{n}\ \sum \limits_{i=1}^n{x}_i $$

### Epigenetic age prediction by pyrosequencing of three CpGs

Pyrosequencing of the three age-associated CpGs was measured in 243 blood samples of healthy controls and 105 [17] and 80 [25] of these were also mentioned in previous work. Furthermore, we analyzed 70 independent AA and 18 independent DKC patients. Pyrosequencing was described in detail before [17] and performed at Cygenia GmbH (Aachen, Germany). In short, DNA was isolated from patient samples with the QIAamp DNA Mini and Blood Mini kit (Qiagen, Hilden, Germany) and bisulfite converted with the EZ DNA Methylation Kit (Zymo Research, Freiburg, Germany). Converted DNA was PCR amplified with the PyroMark PCR kit 800 (Qiagen) and 20 μL PCR product was immobilized to 60 μL Streptavidin Sepharose HP beads (GE Healthcare, Chicago, IL, USA), annealed to 0.8 μL 20 μM sequencing primers for 2 min at 80 °C (see all primer sequences in Additional file 1: Table S4). Samples were measured with the PyroMark Gold Q96 Reagents on a PyroMark Q96 ID and analyzed with the Pyro-Q-CpG 1.0.9 software (all from Qiagen). Epigenetic age was calculated as follows:

Predicted age (in years) = 38.0 − 26.4 × *β* value (cg02228185) − 23.7 × *β* value (cg25809905) + 164.7 × *β* value (CpG upstream of cg17861230)

### MassARRAY analysis of DNA methylation in *PRDM8*

Measurements of all patient data on *PRDM8* and its two CpG sites cg19409579 and cg27242132 have been performed with MassARRAY analysis instead of pyrosequencing. This is due to the fact that the *PRDM8* region has many CpG sites, and pyrosequencing primers overlapping with CpGs were temperature sensitive and did not provide reliable measurements. With MassARRAY, longer amplicons can be measured thus making primer design outside of CpG-rich regions and analysis of these *PRDM8* regions possible. MassARRAY analyses were performed with a MassARRAY Analyzer 4 System (Agena Bioscience, Hamburg, Germany) as previously described [18] at Varionostic GmbH (Ulm, Germany).

### Barcoded bisulfite amplicon sequencing of DNA methylation in *PRDM8*

Two BBA-seq assays were designed around the CpG sites cg27242132 (assay 1) and cg19409579 (assay 2; Additional file 1: Fig. S8) and amplified with the PyroMark PCR kit (Qiagen). The forward and reverse primers contain handle sequences for the subsequent barcoding step (Additional file 1: Tables S5). PCR conditions were 95 °C for 15 min; 40 cycles of 94 °C for 30 s, 53 °C for 30 s, 72 °C for 30 s; and then final elongation at 72 °C for 10 min. The two amplicons of each donor were pooled at equal concentrations, quantified with Qubit (Invitrogen, Carlsbad, CA, USA), and cleaned up with paramagnetic beads from Agencourt AMPure PCR Purification system (Beckman Coulter, Brea, CA, USA). Four microliters of PCR products were subsequently added to 21 μL PyroMark Master Mix (Qiagen) containing 0.4 μM of barcoded primers (adapted from NEXTflexTM 16S V1-V3 Amplicon Seq Kit, Bioo Scientific, Austin, USA) for a second PCR (95 °C for 15 min; 16 cycles of 95 °C for 30 s, 60 °C for 30 s, 72 °C for 30 s; final elongation at 72 °C for 10 min). PCR products were again quantified with the Qubit, combined in equimolar ratios, and cleaned by Select-a-Size DNA Clean & Concentrator Kit (Zymo Research). A 12-pM DNA library was diluted with 15% PhiX spike-in control and eventually subjected to 250 bp paired-end sequencing on a MiSeq lane using the Miseq reagent V2 Nano kit (both from Illumina, San Diego, CA, USA). Obtained FastQ files from the MiSeq were aligned to the reference genome *hg19* using the Bismark tool [60] and DNA methylation values and patterns were extracted with the Bismark methylation extractor. The average number of reads across all samples was 4088 for assay 1 and 3242 for assay 2. For heatmaps, the frequencies of DNA methylation patterns in individual reads were calculated by the number of reads containing the pattern divided by the total number of reads of the target region per sample. The most abundant reads of similar patterns within neighboring CpGs were grouped together and visualization was performed with Python’s package seaborn [61].

### qRT-PCR measurement of patient samples

Total RNA was isolated from 10 healthy controls, 27 AA, and 14 DKC patients with the miRNeasy Kit (Qiagen). One hundred nanograms of RNA was then converted into cDNA with the High Capacity cDNA Reverse Transcription Kit (Thermo Scientific, Waltham, MA, USA). cDNA was analyzed in qPCR with specific primers for either the long transcript of *PRDM8* (NM020226.3), the short transcript of *PRDM8* (NM 001099403.2), or all *PRDM8* transcripts (Primers see in Additional file 1: Table S6) using the Power SYBR Green PCR Master Mix (Thermo Scientific) and the StepOnePlus Real-Time PCR System (Applied Biosystems, Waltham, MA, USA). Data was normalized to GAPDH.

### Generation, cultivation, and genome editing of human iPSCs

Mesenchymal stromal cells were reprogrammed into iPSCs with episomal plasmids and characterized with Epi-Pluri-Score as described before [30, 62]. iPSCs were cultured on tissue culture plastic (TCP) coated with Vitronectin XF (0.5 mg/cm^2^; Stemcell Technologies, Vancouver, Canada) in StemMACS iPS-Brew XF (Miltenyi Biotec, Bergisch Gladbach, Germany). For genome editing, we used a CRISPR/Cas9n double nicking approach [63]. In brief, two pairs of guide RNA (gRNA) were designed targeting the intron/exon boundary at the start codon of the *PRDM8* gene (Fig. 3a, Additional file 1: Table S6). Deletion of the intron/exon boundary leads to a reading frame shift and premature stop codon, thus generating a complete knockout of the PRDM8 protein. gRNA oligonucleotides were cloned individually into a variant of vector pX335 (Addgene #42335, Addgene, Watertown, MA, USA) carrying a Puromycin-GFP selection cassette. gRNA plasmids were transfected into iPSCs using the NEON transfection system (1500 V, 20 ms pulse width, 1 pulse, Thermo Fisher Scientific, Waltham, MA, USA). Transfected cells were selected by puromycin treatment (0.4 μg/mL) for 24 h. Ten days later, individual colonies were picked and screened for deletions in the *PRDM8* target region by PCR (Primers see Additional file 1: Table S7). To further validate pluripotency of iPSCs, we determined DNA methylation at three pluripotency-associated CpGs by pyrosequencing to estimate the Epi-Pluri-Score, as described in detail before [30].

### Embryoid body assay

To test for three-lineage potential of iPSC clones, cells were spontaneously differentiated via the EB assay. iPSCs were incubated with 1 mg/mL collagenase IV (Thermo Fisher Scientific) for 5–15 min and rinsed off with KO-DMEM (Thermo Fisher Scientific). Resulting cell clusters sedimented by gravity at 37 °C for 10 min and were resuspended in EB culture medium containing 20% FCS, 100 μM non-essential amino acids, 2 mM L-glutamine, and 0.2% ß-mercaptoethanol (all Thermo Fisher Scientific) and then transferred to ultra-low attachment plates (Corning, NY, USA) to form EBs in a 3D culture. On day 5, EBs were transferred to plates coated with 0.1% gelatin (Stemcell Technologies) for 2D culture. Cells were cultured for additional 9 days. Gene expression of marker genes was analyzed by quantitative RT-PCR (depicted as mean fold change versus GAPDH). Primer sequences are listed in Additional file 1: Tab. S8.

### Hematopoietic differentiation

Hematopoietic differentiation of human iPSCs was induced with a Spin-EB protocol [31]. iPSCs were cultured on Matrigel Matrix (Corning) and detached as single cells with Accutase (Stemcell Technologies). Three thousand single cells per well were seeded in a U-bottom 96-well plate (Greiner Bio-One, Kremsmünster, Austria) in serum free medium containing 50% IMDM, 50% Ham’s F12, 0.5% BSA, 1% chemically defined lipid concentrate, 2 mM GlutaMAX (all Thermo Fisher Scientific), 400 μM 1-thioglycerol, 50 μg/mL L-ascorbic acid, and 6 μg/mL holo-transferrin (all Sigma Aldrich) supplemented with 10 ng/mL FGF-2 (Peprotech, Hamburg, Germany), 10 ng/mL BMP-4 (Miltenyi Biotec), and 10 μM Y-27632 (Abcam, Cambridge, Great Britain). The plates were centrifuged at 350*g* for 5 min and cells formed EBs on the next day. From day 2 to 16, the EBs were cultured in serum free medium supplemented with 10 ng/mL FGF-2, 10 ng/mL BMP-4, 50 ng/mL SCF (Peprotech), 10 ng/mL VEGF-A (Peprotech), and 10 U/mL penicillin/streptomycin (Thermo Fisher Scientific). Hematopoietic progenitors were harvested after 16 days of differentiation, separated with a 40 μM cell strainer, centrifuged at 200*g* for 5 min, and then used for colony forming unit assay, cytospin, and immunophenotypic analysis.

### Colony forming unit assay

After 16 days of differentiation, 5000 hematopoietic progenitor cells were seeded in 500 μL of methylcellulose-based medium (HSC-CFU lite with EPO; Miltenyi Biotec) in 24-well plates. Colonies were quantified after 12 days.

### Cytospin

Cellular morphology of hematopoietic progenitors was analyzed by cytospins with 1% neutral benzidine (Sigma Aldrich) and Diff Quik staining (Medion Grifols Diagnostics, Düdingen, Switzerland). Cytospins were analyzed with a Leica DMRX microscope (Leica Microsystems, Wetzlar, Germany).

### Immunophenotypic analysis

Flow cytometric analysis of hematopoietic surface markers was performed with a FACS Canto II (BD Biosciences) with the following antibodies: CD3-APC (clone HIT3a), CD31-PE (clone WM59), CD34-APC (clone 581), CD43-FITC (clone 1G10), CD66b-PE (clone G10F5) (all BD Biosciences, Franklin Lakes, USA), CD11c-PE-Cy7 (clone 3.9), HLA-DR-FITC (clone LN3), CD235a-PE (clone HIR2/GA-R2), cKIT-PE-Cy7 (clone 104D2), (all Thermo Fisher Scientific), CD45-APC-Vio770 (clone 5B1), CD33-APC (clone AC104.3E3) (all Miltenyi Biotec), and CD41-PE-Cy7 (clone HIP8; Biolegend, San Diego, CA, USA). Data was analyzed using the FlowJo software (Tree Star, Ashland, OR, USA).

### Neuronal differentiation of iPSCs

Differentiation of iPSCs toward peripheral neurons was performed as previously described [64, 65]. iPSCs were seeded as single cells at a density of 10^5^ cells/cm^2^ in presence of 10 μM Y-27632. At 80–90% confluency, neural conversion was induced by dual-SMAD inhibition: for the first 5 days, LDN-193189 (100 nM or 1 μM, Sigma Aldrich) and SB431542 (10 μM, Miltenyi Biotec) were added to the basal culture medium consisting of knockout DMEM/F-12 containing 15% knockout serum replacement, 1 mM L-glutamine, 100 μM non-essential amino acids, 100 μM β-mercaptoethanol, 100 U/mL penicillin, and 100 μg/mL streptomycin (all Thermo Fisher Scientific). To accelerate neural crest specification, three small molecules (3 μM CHIR99021, 10 μM DAPT, and 10 μM SU5402, all Tocris, Bristol, UK) were added between days 2 to 10. After 4 days, the medium was supplemented in increasing percentages with DMEM/F-12, containing 10 ml/L N2, 20 ml/L B27 minus vitamin A supplements and 100 U/mL penicillin, 100 μg/mL streptomycin (all Thermo Fisher Scientific). N2/B27 medium was added to the basal medium at 25% between days 4 and 5, 50% between days 6 and 7, and 75% between days 8 and 10. On day 10, cells were dissociated using Accutase and seeded on glass coverslips coated with 50 μg/mL Poly-L-Ornithine (Sigma Aldrich) and 5 μg/mL Laminin (Thermo Fisher Scientific) and further cultured for up to 3 weeks with N2/B27 medium supplemented with 20 ng/mL NGF (R&D Systems, Minneapolis, MN, USA), BDNF, GDNF (all PeproTech), and 200 ng/mL L-ascorbic acid (Sigma Aldrich). Medium was changed every 3–4 days.

### DNA methylation and gene expression analyses

DNA methylation analysis of samples after 10 days of neural differentiation was performed with the Infinium MethylationEPIC BeadChip (Illumina). Therefore, DNA was isolated with the NucleoSpin Tissue kit (Macherey-Nagel, Düren, Germany) and bisulfite conversion as well as hybridization was performed at Life and Brain GmbH (Bonn, Germany). Data was preprocessed with the Bioconductor Illumina Minfi package for R [66–72] and normalized with quantile normalization. Data of DNA methylation profiles has been deposited at Gene Expression Omnibus (GEO) under the reference ID GSE141106. For differential DNA methylation analysis, CpGs in X and Y chromosomes as well as SNPs were excluded and we then filtered for beta-values with a difference between *PRDM8*^*−/−*^ and WT of <*−* 0.2 or > 0.2.

RNA-Seq of samples after 27 days of neural differentiation was performed at Life and Brain GmbH (Bonn, Germany) on a HiSeq 2500 sequencer (Illumina). Adapter trimming and rRNA removal in raw fastq files was performed using TrimGalore (Babraham Bioinformatics, Cambridge, UK) and SortMeRNA [73], respectively. Reads were aligned to the human genome build GRCh38 with STAR aligner [74]. Read counts were retrieved by HTSeq-count and further analyzed with the R package DESeq2. RNA-Seq data has been deposited at Gene Expression Omnibus (GEO) under the reference ID GSE141107. Gene ontology enrichment analyses of differentially expressed genes were performed using the PANTHER software [75]. Categories with more than 1000 genes were excluded and similar categories are only listed once.

### Additional datasets of premature aging syndromes

We utilized 450 k Illumina Bead Chip datasets of DKC (GSE75310 [18]), Down syndrome (GSE52588 [34, 76, 77]), Werner syndrome (GSE42865 [78]), Hutchinson-Gilford-Progeria (GSE42865), and healthy controls (GSE36054 [79], GSE32148 [80], GSE49064 [81]) to analyze DNA methylation patterns within the *PRDM8* gene. Gene expression analyses for *PRDM8* were performed with the following available datasets with corresponding controls: Down syndrome (GSE35665 [26]), Werner syndrome (GSE48761 [27]), and HGPS (GSE69391 [28], GSE3860 [29]).

### Statistical analysis

Statistical significance of delta telomere age, delta epigenetic age, methylation values of specific CpG sites, and absolute cell counts were estimated by unpaired *t* tests. CFU colonies and absolute cell counts of iPS-derived hematopoietic progenitor cells are presented as means ± standard deviation.

## Supplementary information

**Additional file 1: **Combined PDF of Supplemental Figures S1 – S6 and Supplemental Tables S3 – S4. Fig. S1: Telomere age of lymphocytes in dyskeratosis congenita and aplastic anemia. Fig. S2: The CpG site cg19409579 in *PRDM8* is hypermethylated in AA and DKC. Fig. S3: Aberrant DNA methylation in *PRDM8* is not correlated with blood counts. Fig. S4: Aberrant DNA methylation patterns of *PRDM8* in premature aging diseases. Fig. S5: Characterization of *PRDM8* knockout induced pluripotent stem cells. Fig. S6: Colony forming unit assay of iPSC-derived hematopoietic progenitor cells. Fig. S7: Gene ontology analysis after neuronal differentiation. Fig. S8: Genomic regions for the BBA-seq assays within *PRDM8*. Tab. S4: Primers used for pyrosequencing of the epigenetic aging signature. Tab S5: Primers for bisulfite barcoded amplicon sequencing of *PRDM8*. Tab. S6: Quantitative RT-PCR primers used for *PRDM8* gene expression analysis. Tab. S7: Guide RNAs used for creating CRISPR knockouts of *PRDM8* in iPSCs. Tab. S8: Quantitative RT-PCR primers used for analysis of embryoid body assays.

**Additional file 2: **Tab. S1: Differentially expressed genes in *PRDM8*^*-/-*^ *versus* control. Genes that are either four-fold downregulated (1280 CpG sites) or upregulated (1769 CpG sites; log2 fold change <-2 or > 2) in *PRDM8*^*-/-*^ compared to control iPSCs with ENSEMBL ID, reads per million (RPM) of the knockout and control cells, and log2 fold change of knockout and control.

**Additional file 3: **Tab. S2: Differentially methylated CpG sites in *PRDM8*^*-/-*^ *versus* control. CpG sites that are either hypermethylated (1511 CpG sites) or hypomethylated (1, 738 CpG sites; delta β-value >0.2 or < -0.2) in *PRDM8*^*-/-*^ compared to control iPSCs with β-values of the knockout and control cells, and the difference in methylation between knockout and control.

**Additional file 4:.** Tab. S3: Patient data of all DKC, AA samples and healthy controls. List of all patient samples and healthy controls with their according gender, chronological age, diagnosis, mutated genes, and blood counts.

## Data Availability

Data of DNA methylation profiles has been deposited at Gene Expression Omnibus (GEO) under the reference ID GSE141106. RNA-Seq data has been deposited at GEO under the reference ID GSE141107.

## Abbreviations

AA: Aplastic anemia
BBA-seq: Barcoded bisulfite amplicon sequencing
CpG site: CG dinucleotide
DKC: Dyskeratosis congenita
EB: Embryoid body
gRNA: Guide RNA
HGPS: Hutchinson-Gilford-Progeria syndrome
iPSC: Induced pluripotent stem cell
MAE: Mean absolute error
qRT-PCR: Quantitative reverse transcription PCR
TCP: Tissue culture plastic
