# Supplementary material for: PRDM8 reveals aberrant DNA methylation in aging syndromes and is relevant for hematopoietic and neuronal differentiation
Source: Clin Epigenetics. 2020 Aug 20;12:125. doi: 10.1186/s13148-020-00914-5 (PMC7439574; doi:10.1186/s13148-020-00914-5)
Supplement: Supplementary file 1 — Additional file 1: Combined PDF of Supplemental Figures S1 – S6 and Supplemental Tables S3 – S4. Fig. S1: Telomere age of lymphocytes in dyskeratosis congenita and aplastic anemia. Fig. S2: The CpG site cg19409579 in PRDM8 is hypermethylated in AA and DKC. Fig. S3: Aberrant DNA methylation in PRDM8 is not correlated with blood counts. Fig. S4: Aberrant DNA methylation patterns of PRDM8 in premature aging diseases. Fig. S5: Characterization of PRDM8 knockout induced pluripotent stem cells. Fig. S6: Colony forming unit assay of iPSC-derived hematopoietic progenitor cells. Fig. S7: Gene ontology analysis after neuronal differentiation. Fig. S8: Genomic regions for the BBA-seq assays within PRDM8. Tab. S4: Primers used for pyrosequencing of the epigenetic aging signature. Tab S5: Primers for bisulfite barcoded amplicon sequencing of PRDM8. Tab. S6: Quantitative RT-PCR primers used for PRDM8 gene expression analysis. Tab. S7: Guide RNAs used for creating CRISPR knockouts of PRDM8 in iPSCs. Tab. S8: Quantitative RT-PCR primers used for analysis of embryoid body assays. [file 13148_2020_914_MOESM1_ESM.pdf]

Supplemental Data

PRDM8 reveals aberrant DNA methylation in aging syndromes and is relevant for hematopoietic and neuronal differentiation

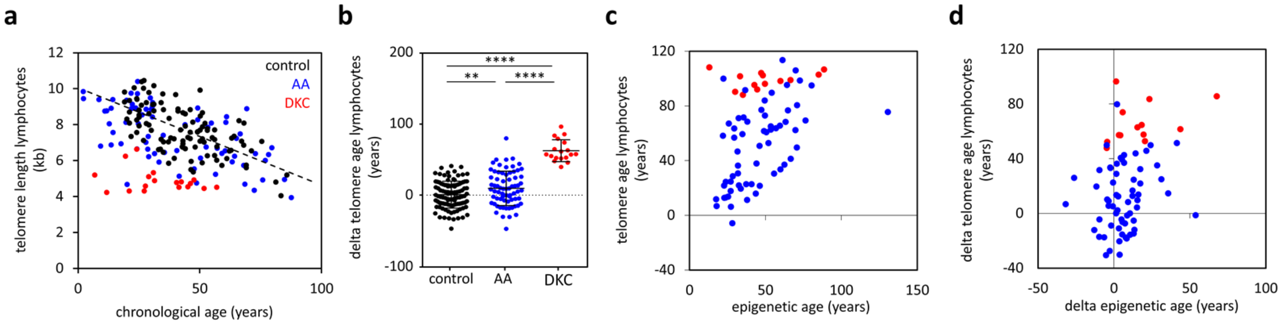

Fig. S1: Telomere age of lymphocytes in dyskeratosis congenita and aplastic anemia.

(a) Telomere lengths of 105 healthy donors (1), 70 aplastic anemia (AA) and 18 dyskeratosis congenita (DKC) patients measured in lymphocytes were correlated to chronological age (there are more samples than for granulocytes (Fig 1a), because telomere length in granulocytes was not always measured). DKC and AA patients show a reduced telomere length compared to healthy controls. **(b)** The difference between predicted telomere age and chronological age were higher for DKC and AA patients than for healthy controls. T-test: ** $P < 0.001$, *** $P < 0.0001$. **(c,d)** Telomere age and epigenetic age (c), as well as delta telomere age and delta epigenetic age (d) are not correlated.

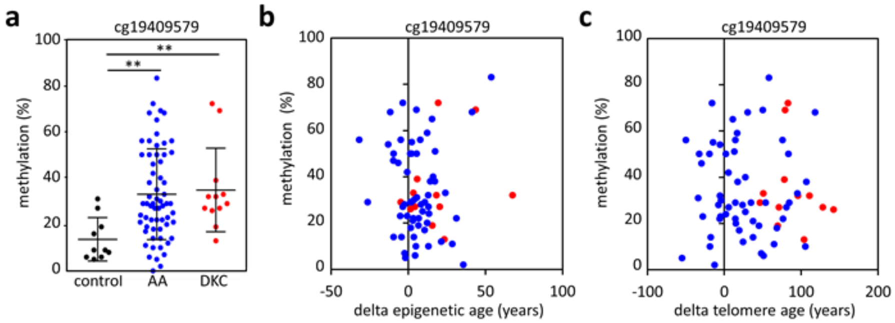

Fig. S2: The CpG site cg19409579 in *PRDM8* is hypermethylated in AA DKC.

(a) DNA methylation at the CpG site cg19409579 is significantly increased in 62 aplastic anemia (AA) and 12 dyskeratosis congenita (DKC), as compared to 10 healthy controls (2). T-test: ** $P < 0.01$. **(b,c)** DNA methylation in cg19409579 does not correlate to epigenetic age (b) or telomere age (c).

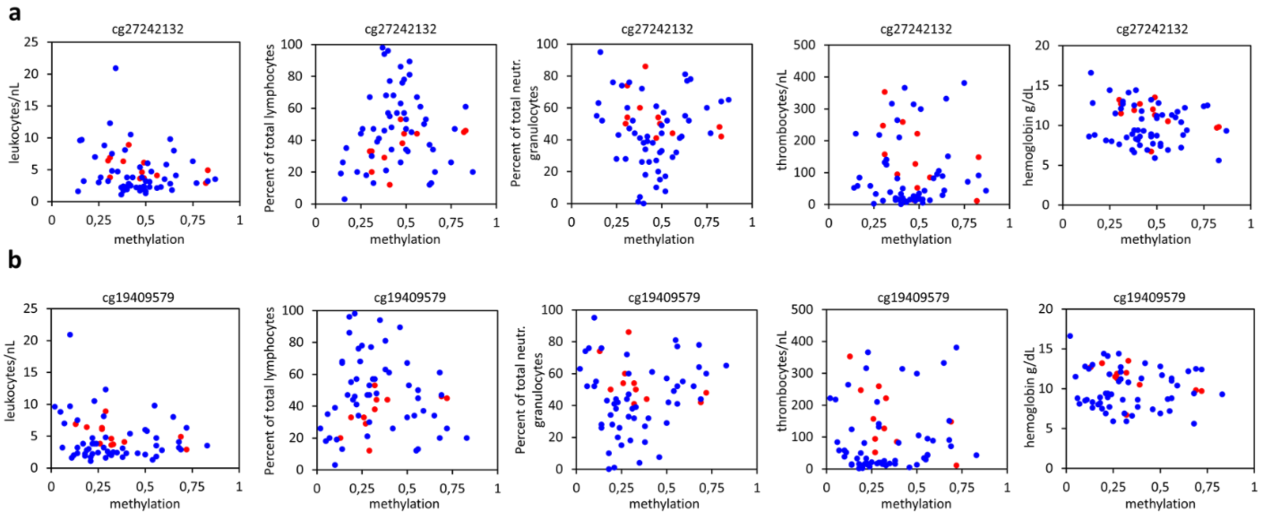

Fig. S3: Aberrant DNA methylation in *PRDM8* is not correlated with blood counts.

The DNA methylation levels in the two *PRDM8* associated CpGs (**a**: cg27242132; **b**: cg19409579**)** were analyzed in 56 aplastic anemia (AA) and 12 dyskeratosis congenita (DKC) patients in comparison to different blood counts (Blood counts were not available for all patient samples). There was no clear correlation.

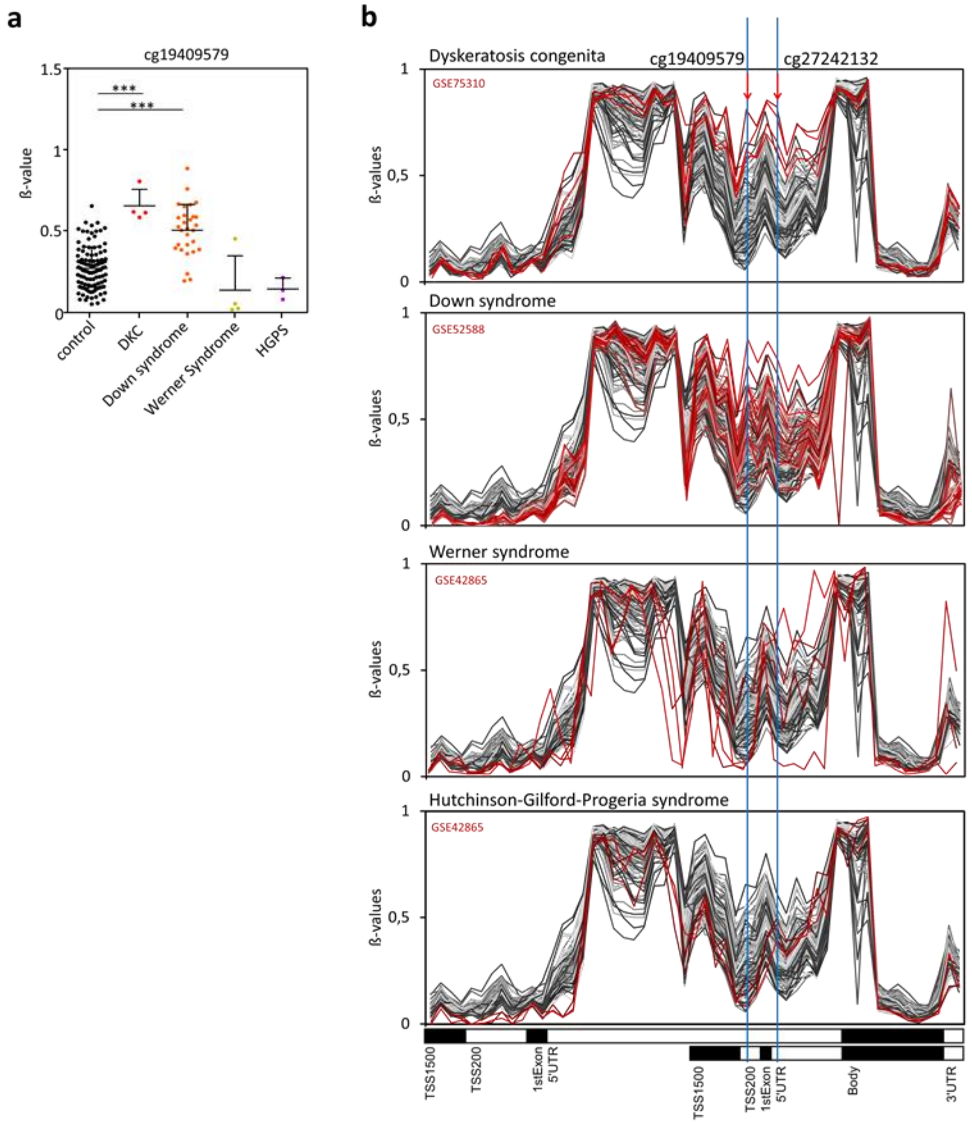

Fig. S4: Aberrant DNA methylation patterns of *PRDM8* in premature aging diseases.

(a) DNA methylation (β-values) at the *PRDM8* associated CpG site (cg19409579) in 103 healthy controls (GSE36054, GSE32148, GSE49064), 4 DKC samples (GSE75310), 29 Down syndrome samples (GSE52588), 4 Werner syndrome samples (GSE42865) and 3 Hutchinson-Gilford-Progeria Syndrome (HGPS) samples (GSE42865). T-test: *** P < 0.0001. (**b**) DNA methylation levels (β-values) at all CpG sites associated with *PRDM8* show hypomethylation in the promoter region of the long transcript (NM_020226.3) and hypermethylation at the CpG sites cg19409579 and cg27242132 (position highlighted by the blue line) in the *PRDM8* gene in premature aging syndromes compared to healthy controls. Shown in grey are 103 healthy controls (GSE36054, GSE32148, GSE49064), shown in red are the diseased samples as mentioned above.

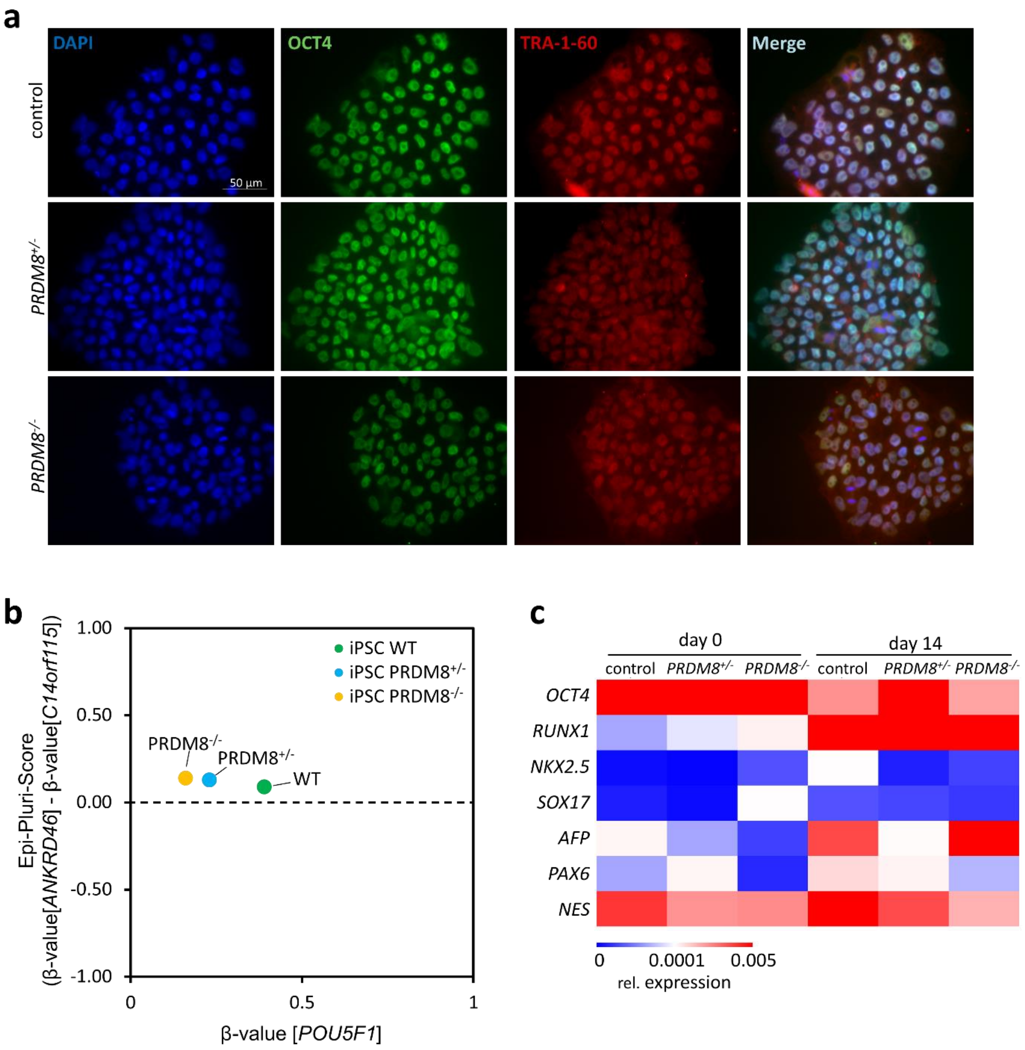

Fig. S5: Characterization of *PRDM8* knockout induced pluripotent stem cells.

(a) Immunofluorescence microscopy of induced pluripotent stem cells (iPSCs) for the pluripotency markers OCT4 and TRA-1-60. Nuclei were counterstained with DAPI. All clones are positive for both pluripotency markers. **(b)** To further validate pluripotency of the iPSC clones, we analyzed pluripotency associated CpGs by pyrosequencing to determine the Epi-Pluri-Score (3). All clones revealed positive Epi-Pluri-Score and hypomethylation in the *POU5F1* promotor, indicating that they were pluripotent. **(c)** Quantitative RT-PCR analysis of marker genes for ectodermal, mesodermal, and endodermal lineages was performed at day 0 (iPSCs) and after 14 days of differentiation in EBs. *OCT4* is used as a pluripotency marker, *PAX6* and *NES* as ectodermal markers, *SOX17* and *AFP* as endodermal markers, and *RUNX1* and *NKX2.5* as mesodermal markers. Color code depicts mean fold change *versus GAPDH*. Notably, upregulation of the neuronal markers nestin (*NES*) and paired box 6 (*PAX6*) is reduced in *PRDM8*$^{+/-}$ and *PRDM8*$^{-/-}$.

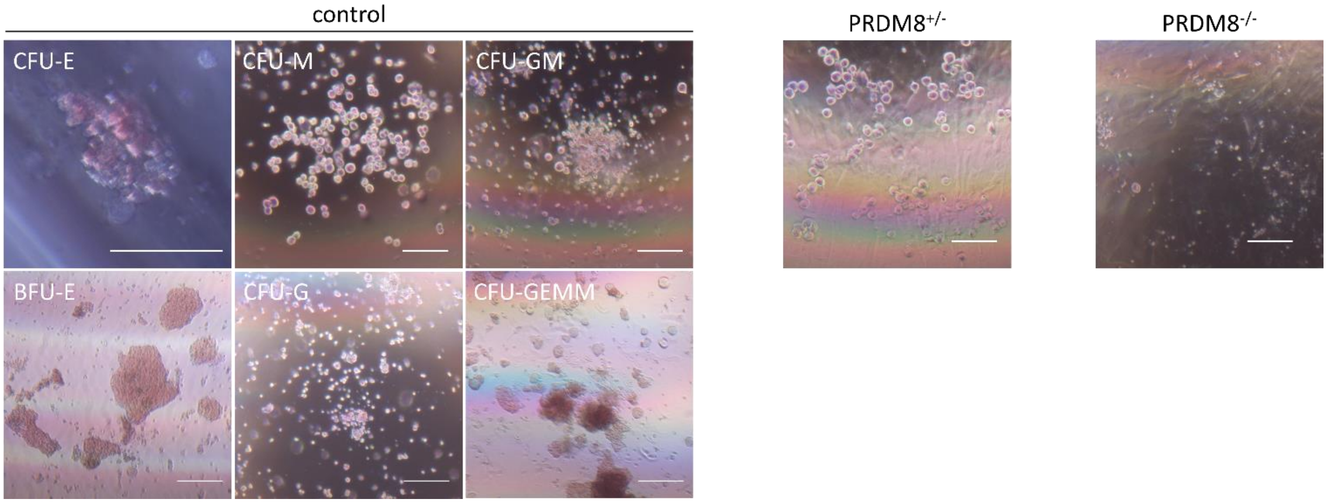

Fig. S6: Colony forming unit assay of iPSC-derived hematopoietic progenitor cells.

Representative phase contrast pictures of colony forming units (CFUs) of control iPSCs with typical morphology. Knockout clones show impaired differentiation with no CFU colonies in *PRDM8*$^{-/-}$ and only few CFU-M in *PRDM8*$^{+/-}$. CFU-E: Colony Forming Unit Erythroid; BFU-E: Burst Forming Unit Erythroid; CFU-M: Colony Forming Unit Macrophage; CFU-G: Colony Forming Unit Granulocyte; CFU-GM: Colony Forming Unit Granulocyte, Macrophage; CFU-GEMM: Colony Forming Unit Granulocyte, Erythrocyte, Macrophages, Megakaryocyte. Scale bar: 200 µm.

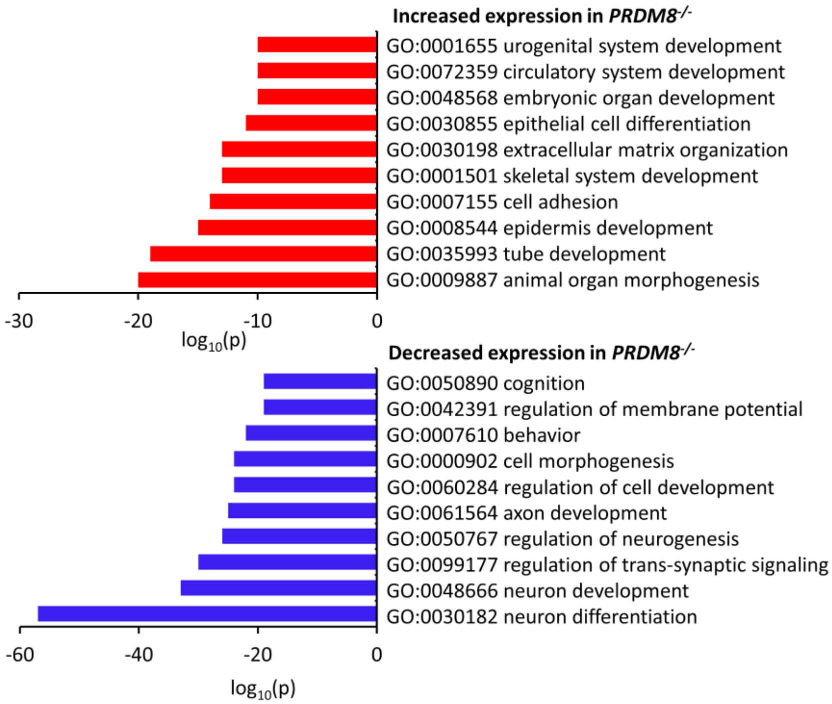

Fig. S7: Gene ontology analysis after neuronal differentiation.

Gene ontology analysis of genes with at least four-fold differential expression (log2 FC > 2 or < -2) in knockout *versus* control cells revealed enrichment in cell adhesion or developmental processes and decreased representation of genes for neural activity in *PRDM8*$^{-/-}$ clones.

PRDM8-1 (chr4:80,197,876-80,198,069)

TTCTTGAGGGGCTGTCTACTGCCAGTAATATTGTACAAAAGGAGGAG**CG**GGTGGTGAGCACTTTGC**CG**GAG
48 67

AAGC**CG**GAGACTGGGGAG**CGCGCG**GC**CG**CCAGACAATGCCTGCCTAGAGGGA**CG**GGCCAGTGG**CG**GGCA
76 90 92 94 98 124 135

GCAG**CG**AC**CG**GCTTTCAGAAAGCCC**CG**C**CG**GCTCTGGGGTATATTCCCAGGGCA
145 149 166 169

PRDM8-2 (chr4:80,197,190-80,197,431)

GGGGAAAGGCCCTCTCCAATCATTTTGGTACCTTCCTTTCCAATCCTGAAA**CG**ATTTTCCCCTCCTAGCAGTC
52

CCAGAGGGCC**CG**AGCTTTAGGAGGAGG**CG**GGC**CG**GGA**CG**CTCTCTGAGAGG**CG**TTGCCACC**CG**GGGTAG
84 101 106 111 125 135

G**CG**CTCAGGATCTG**CG**GGG**CGCG**GGCAGGC**CG**GGGGAAAAAGCTG**CG**AGGCAGG**CG**G**CG**C**CG**GAGGGA
144 157 162164 173 188 197 200 203

G**CG**CCTGGCCCAGCAAAGAGTTAAAGGGAGGG
212

Fig. S8: Genomic regions for the BBA-seq assays within *PRDM8*.

Sequences for two genomic regions of *PRDM8* (assay 1 and assay 2) that were analyzed by bisulfite barcoded amplicon sequencing (BBA-seq) are depicted and all CpG sites are highlighted in red. The CpGs are indicated by their ordering number in the sequences. The underlined CpGs are cg27242132 and cg19409579.

Supplemental Tables

Table S1: Differentially expressed genes in *PRDM8*$^{-/-}$ *versus* control

This table is provided as Additional File 2

Table S2: Differentially methylated CpG sites in *PRDM8*$^{-/-}$ *versus* control

This table is provided as Additional File 3

Table S3: Patient data of all DKC, AA samples and healthy controls

This table is provided as Additional File 4

Table S4: Primers used for pyrosequencing of the epigenetic aging signature.

Primer	Sequence (5' – 3')
ASPA Fw	biotin-ATTATTTGGTGAAATGATT
ASPA Rv	CAACCCTATTCTCTAAATCTC
ASPA seq	CCCTATTCTCTAAATCTCA
ITGA2B Fw	biotin-TAATTTTTTTTGGGTGATG
ITGA2B Rv	ACCAAAAATAAACAATATACTCAAT
ITGA2B seq	CAATATACTCAATACTATACCT
PDE4C Fw	AGGTTTGTAGTAGGTTGAG
PDE4C Rv	biotin-AACTCAAATCCCTCTC
PDE4C seq	GTTATAGTATGATTAGAGTTT

Table S5: Primers for bisulfite barcoded amplicon sequencing of PRDM8

Primer	Sequence (5' – 3')
PRDM8-1 Fw	CTCTTTCCCTACACGACGCTCTTCCGATCTTTTTTGAGGGGTTGTTTATTGTTAGT
PRDM8-1 Rv	CTGGAGTTCAGACGTGTGCTCTTCCGATCTTACCCTAAAAATATACCCCAAAACC
PRDM8-2 Fw	CTCTTTCCCTACACGACGCTCTTCCGATCTGGGGAAAGGTTTTTTTTAATTATTTT
PRDM8-2 Rv	CTGGAGTTCAGACGTGTGCTCTTCCGATCTCCCTCCCTTTAACTCTTTACTAAACC

Table S6: Quantitative RT-PCR primers used for *PRDM8* gene expression analysis.

Primer	Sequence (5' – 3')
NM020226.3 Fw	ACGAGCAAATGCGATCTCTG
NM020226.3 Rv	ATGCTTCCCAGGCTGATTTGA
NM001099403.2 Fw	TCTCCCCATCTCTCCATCTC
NM001099403.2 Rv	GATGCCAGTATCCTCCATCA
PRDM8_all Tr. Fw	ACCAGCGTTTACACCACCTG
PRDM8_all Tr. Rv	CCATTTGCTGCTGAGGTGTC
GAPDH Fw	GAAGGTGAAGGTCGGAGTC
GAPDH Rv	GAAGATGGTGATGGGATTTC

Table S7: Guide RNAs used for creating CRISPR knockouts of *PRDM8* in iPSCs.

Primer	Sequence (5' – 3')
PRDM8 1a Fw	AAACTCTTGTAGGCTGCAGAGAAAC
PRDM8 1a Rv	CACCGTTTCTCTGCAGCCTACAAGA
PRDM8 1b Fw	CACCGGTTAGCTGACACAGAATTAG
PRDM8 1b Rv	AAACCTAATTCTGTGTCAGCTAACC
PRDM8 2a Fw	AAACGCGTTTACACCACCTGCGACC
PRDM8 2a Rv	CACCGGTCGCAGGTGGTGTAAACGC
PRDM8 2b Fw	CACCGATCCCTGAGAATGCTATATT
PRDM8 2b Rv	AAACAATATAGCATTCTCAGGGATC

Table S8: Quantitative RT-PCR primers used for analysis of embryoid body assays.

Primer	Sequence (5' – 3')
AFP Fw	GCCAAGCTCAGGGTGTAG
AFP Rv	CAATGACAGCCTCAAGTTGT
Nav1.7 Fw	CACAATCCCAGCCTCACAGT
Nav1.7 Rv	CTGAGGAGCTTGACCGGTTTA
NEFH Fw	CGACATTGCCTCCTACCAG
NEFH Rv	TCCGACACTCTTCACCTTCC
NES Fw	CCTCAAGATGTCCCTCAGCC
NES Rv	CCAGCTTGGGGTCCTGAAAG
NXK2.5 Fw	ACCTCAACAGCTCCCTGACTCT
NXK2.5 Rv	ATAATCGCCGCCACAAACTCTCC
OCT4 Fw	GGGGGTTCTATTTGGGAAGGTA
OCT4 Rv	ACCCACTTCTGCAGCAAGGG
PAX6 Fw	CAGACACAGCCCTCACAAAC
PAX6 Rv	TCATAACTCCGCCCATTCAC
PRDM8 Fw*	ACCGTATATCTTTCGGGTAGACA
PRDM8 Rv	CTAGAGGGGCAGAGCAAGAG
RUNX1 Fw	CCGAGAACCTCGAAGACATC
RUNX1 Rv	GTCTGACCCTCATGGCTGT
SOX1 Fw	CCTGTGTGTACCCTGGAGTTTCTGT
SOX1 Rv	TGCACGAAGCACCTGCAATAAGATG
TAC1 Fw	CACAATCCCAGCCTCACAGT
TAC1 Rv	CAAAGAACTGCTGAGGCTTG

* Primer anneals to the deleted region in the *PRDM8* transcript.

Supplemental References

1. Weidner CI, Lin Q, Koch CM, Eisele L, Beier F, Ziegler P, et al. Aging of blood can be tracked by DNA methylation changes at just three CpG sites. Genome Biol. 2014;15(2):R24.

2. Weidner CI, Lin Q, Birkhofer C, Gerstenmaier U, Kaifie A, Kirschner M, et al. DNA methylation in PRDM8 is indicative for dyskeratosis congenita. Oncotarget. 2016;7(10):10765-72.

3. Lenz M, Goetzke R, Schenk A, Schubert C, Veeck J, Hemeda H, et al. Epigenetic biomarker to support classification into pluripotent and non-pluripotent cells. Sci Rep. 2015;5:8973.
